# Proteomic Changes between Male and Female Worms of the Polychaetous Annelid *Neanthes arenaceodentata* before and after Spawning

**DOI:** 10.1371/journal.pone.0072990

**Published:** 2013-08-30

**Authors:** Kondethimmanahalli H. Chandramouli, Timothy Ravasi, Donald Reish, Pei-Yuan Qian

**Affiliations:** 1 KAUST Global Collaborative Research, Division of Life Science, Hong Kong University of Science and Technology, Hong Kong SAR, China; 2 Integrative Systems Biology Laboratory, Division of Biological and Environmental Sciences and Engineering, Division of Applied Mathematics and Computer Sciences, King Abdullah University of Science and Technology, Thuwal, Kingdom of Saudi Arabia; 3 Department of Biological Sciences, California State University Long Beach, California, United States of America; National Center for Biotechnology Information (NCBI), United States of America

## Abstract

The *Neanthes* *acuminata* species complex (Polychaeta) are cosmopolitan in distribution. *Neanthesarenaceodentata* , Southern California member of the *N* *. acuminata* complex, has been widely used as toxicological test animal in the marine environment. Method of reproduction is unique in this polychaete complex. Same sexes fight and opposite sexes lie side by side until egg laying. Females lose about 75% of their weight and die after laying eggs. The male, capable of reproducing up to nine times, fertilizes the eggs and incubates the embryos for 3-4 weeks. The objective of this study was to determine if there is any set of proteins that influences this unique pattern of reproduction. Gel-based two-dimensional electrophoresis (2-DE) and gel-free quantitative proteomics methods were used to identify differential protein expression patterns before and after spawning in both male and female *N* *. arenaceodentata* . Males showed a higher degree of similarity in protein expression patterns but females showed large changes in phosphoproteme before and after spawning. There was a decrease (about 70%) in the number of detected phosphoproteins in spent females. The proteins involved in muscular development, cell signaling, structure and integrity, and translation were differentially expressed. This study provides proteomic insights of the male and female worms that may serve as a foundation for better understanding of unusual reproductive patterns in polychaete worms.

## Introduction

Polychaete species are widely distributed in the marine environment and inhabit estuaries to deep-hydrothermal vents [1]. Members of the polychaete *Neanthes* *acuminata* species complex are cosmopolitan in distribution [2,3]. Populations from the east coast of North America are referred to as *N* *. acuminata* ; species from Europe are known as *N* *. caudata* ; southern California as *N* *. arenaceodentata* ; and Asia *as* *N* *. crigognatha* . All are morphologically identical and have the same reproductive characteristics [3]. The laboratory populations of *N* *. arenaceodentata* have been used in many studies: toxicity [4,5], reproductive behavior [3], vitellogensis [6], feeding rate metabolism [7] and nervous system architecture [8]. The reproductive behavior is unusual and unique in the genus [2]. Same sexes fight and opposite sexes lie side by side within a mucoid tube until female lays her eggs. The female dies after laying eggs, and the male incubates the fertilized eggs for three to four weeks. During embryonic development the male undulates his body within his tube which insures clean water and expelling metabolic wastes. Males will fight off both sexes during the time period of incubation. About 30% of the time the male will eat the embryos or developing larvae which is probably the result of disturbance or insufficient food. The body muscles of the female during the process of gametogenesis are reabsorbed and supply the material for the large yolky eggs (500-600 µm in diameter). Females who have laid eggs are referred to as spent females. Her locomotive ability is reduced after egg laying; her digestive tract is complete but she is unable to protrude her proboscis to feed because of reduced musculature and may either leave the tube or be eaten by the male [2]. Males are capable of reproducing up to 9 times [4]. Given the uniqueness of *N* *. arenaceodentata* ’s behavior and reproduction, the objective of this research is to characterize the proteome and protein modification (phosphorylation) dynamics to determine why the female can only reproduce once and the male many times.

In recent years, proteomic analyses have been used to compare proteome patterns in polychaetes and other worm species. Previously we reported changes in patterns of proteins and phosphoproteins during early larval development of *N* *. arenaceodentata* [9]. OMIC analysis also provided information on genes/proteins and molecular pathways during larval metamorphosis of the polychaete *Pseudopolydoravexillosa* [10]. Proteomic analysis revealed invasion and habitat adaptation success in two closely related polychaetes *Marenzelleria* *neglecta* and *Marenzelleria* *viridis* [11]. Transcriptome and proteome analysis provided insights in understanding metal resistance in *Ophelina* sp. [5]. In other species, proteomic techniques resulted in the identification of proteins during reproductive development. For example, identification of differentially expressed proteins provided insights into the development in the male and female trematode *Schistosoma* *japonicum* [12]. Tissue-specific proteins were identified in the male reproductive system of *Drosophila melanogaster* [13] and stage- and gender-specific proteins in the nematode *Brugia malayi* [14].

The objective of this study is to identify signature proteins during the reproductive period of male and female *N* *. arenaceodentata* . Gel-based analysis indicated that the proteome abundance was highly conserved in male worms but significantly different in spent female worms. Furthermore, owing to limitations of two-dimensional gel electrophoresis (2-DE), gel-free proteomics was used to determine quantitative proteomic differences between male and females.

## Materials and Methods

### Specimen culture

**Figure 1:**
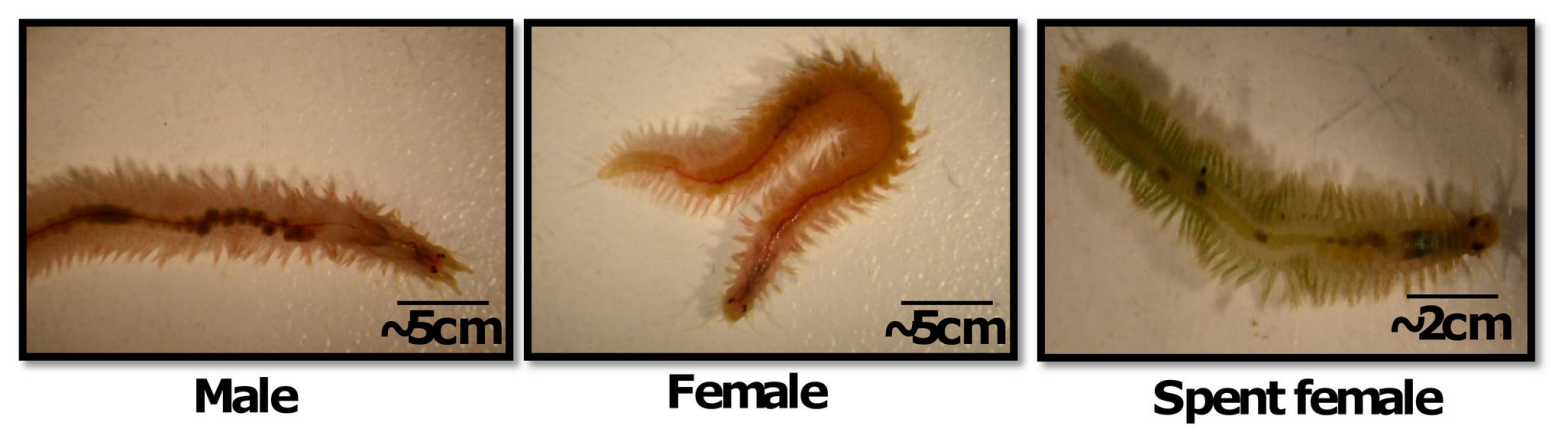
Reproductive stages of the *Neanthesarenaceodentata* . Four stages were chosen for proteomic analysis: mature virgin males (MM), males after first spawning (MAS), females with maturing ova (MF), and spent females (SF).

Living *Neanthesarenaceodentata* were collected from Los Angeles Harbor, USA in 1964. A population was established then and has undergone over 200 generations in the laboratory. All descendants are derived from the original collection of six animals. The following life cycle stages were used in this study: mature virgin males (MM), males after first spawning (MAS), females with maturing ova (MF), and spent females (SF) (Figure 1). A minimum of 10 worms of each stage were frozen and shipped in dry ice by overnight express to Hong Kong. Worms were thawed and washed in seawater to remove debris.

### 2-DE and image analysis

The protein extraction and 2-DE was performed as described by Chandramouli et al. [11]. Worms were transferred to lysis buffer (7 M urea, 2 M thiourea, 4% [3-[(3 cholamidopropyl) dimethylammonio]-1- propanesulfonate] (CHAPS), 40 mM dithiothreitol (DTT) and protease inhibitors (Roche Applied Science, Mannheim, Germany) and then sonicated on ice using 10 sec blasts of 15% amplitude. The homogenate was centrifuged at 13,000 rpm for 15 min and the supernatant protein was purified using a 2-DE cleanup kit (Bio-Rad, Hercules, CA, USA). The protein pellets were solubilized in lysis buffer and quantified using an RC-dC kit (Bio-Rad, Hercules, CA, USA). Three hundred µg of each sample were rehydrated in a buffer (7M of urea, 2M of thiourea, 4% CHAPS, 40mM of DTT, 0.5% pI 4-7 ampholyte, and 1% bromophenol blue) on 17 cm immobilized pH gradient (IPG) strips (pH 4-7) for 14 hr. Isoelectrical focusing (IEF) was performed at 250 V for 20 min, followed by a gradient from 1,000 to 8,500V to reach a total of 60,000Vh on Protean IEF Cell (Bio-Rad, Hercules, CA, USA). The 2-DE gels were fixed for 1 hr in a mixture of 40% methanol and 10% acetic acid and stained for phosphoproteins using ProQ Diamond dye (Invitrogen, Eugene, OR, USA) for 3 hr (1 hr intervals of three times). The gels were washed and scanned using a Typhoon trio imager (GE Healthcare, Piscataway, NJ, USA). These gels were then stained for total proteins using Sypro Ruby dye (Invitrogen, Eugene, OR, USA) and scanned using the Typhoon trio imager. Quantitative and qualitative data for protein spots of three biological replicate gels were obtained by PDQuest software (Bio-Rad, Hercules, CA, USA) as described by Thiyagarajan et al. [15]. Spot detection in each gel was verified by manual inspection of spots to obtain a similar pattern of spots for analysis.

### Dephosphorylating phosphoprotein spots by phosphatase treatment

Protein was extracted from mature worms and phosphatase treated as described in Chandramouli et al. [11]. Two phosphatase (λ-PPase) treated (New England Bio labs, Ipswich, MA, USA) and untreated protein samples were incubated overnight at 30^0^C. The samples were purified using 2-DE cleanup kit and and then analyzed using 2-DE method as described above. The gels were stained for phosphoproteins using ProQ Diamond dye and scanned using a Typhoon trio imager.

### Mass spectrometry

The differentially expressed or abundant stage-specific protein spots were excised and digested in 20 µl of 12.5 ng/ml trypsin (Promega, Madison, WI, USA) for 16 hr at 37^o^C. The peptides were extracted, dried, reconstituted in 15 µl of 0.1% formic acid, and purified using C18 ZipTips (Millipore, Bellirica, MA, USA). The purified dried peptides were subjected to nanoflow UPLC (nanoAcquity, Waters, Milford, MA, USA) coupled with an ESI-hybrid Q-TOF (Premier, Waters, Milford, MA, USA) tandem mass spectrometer as described in Zhang et al. [16]. The instrument was set to positive ion data-dependent acquisition mode with smass range of 300-1600 m/*z*. Three abundant peptides with +2 to +4 charge were selected for MS/MS analysis. Protein identification was achieved using the in-house built transcriptome database of the polychaete *Hydroides* *elegans* *.*

### Quantitative proteomics: Protein extraction, peptides labeling and fractionation

Sample preparation for quantitative proteomics was carried as described by Chandramouli et al. [10] with modifications. The proteins from MM and MF worms were solubilized in 8M urea and quantified using an RC-dC kit (Bio-Rad, Hercules, CA, USA). Acetone precipitated protein samples were reduced and alkylated with reagents supplied in the isobaric tags for relative and absolute quantitation (iTRAQ) kit (Applied Biosystems, Foster City, CA, USA). One hundred twenty µg of protein from each sample were digested with 1:40 enzyme to protein mass ratio of trypsin gold (Promega, Madison, WI, USA) overnight at 37^o^C. The protein digests were desalted using Sep-Pak C18 cartridges (Waters, Milford, MA, USA) and dried under vacuum using a Speed Vac (Thermo Electron, Waltham, MA, USA). Peptides were labeled with the iTRAQ reagents according to the manufacturer’s recommendations. MM peptides were labeled with tags 114 or 115 and MF peptides were tagged with 116 or 117 reporters respectively. A peptide mixture (for a total of about 400 µg of peptide digests) was made with all four tagged samples and desalted using Sep-Pak C18 cartridges to remove the detergents and excess iTRAQ reagents. Peptides were fractionated using a 3100 OFFGEL Fractionator by following manufacture guidelines (Agilent Technologies, Böblingen, Germany). 50 to 150 µl of fractions were recovered for each well and low peptide fractions from acidic (fractions 1-3) and basic (fractions 22-24) pI ranges were pooled. Peptides were then desalted in a Sep-Pak C18 cartridge, dried using a vacuum concentrator and resuspended in 10 µl of 0.1% formic acid. LC-MS/MS was performed as described above for 2-DE protein samples using a nanoflow UPLC (nanoAcquity, Waters) and ESI-hybrid Q-TOF (Premier, Waters, Milford, MA USA).

### Protein identification and quantification

Protein identification and quantification followed procedures described in [10,17]. Micromass peak lists (pkl) containing the values from more than one spectrum are generated by a mass spectrometer’s data handling system. A python script was applied to extract the reporter mass, i.e. 114-118 Da, from the non-deisotoping and deisotoping pkl files. The combined mass spectra pkl files were merged and searched against the concatenated “target” (real sequences of *H. elegans* (Hydroides) transcriptome) and “decoy” (reversed sequences of *H. elegans* transcriptome) translated sequences through search engine MASCOT version 2.3.0 (Matrix Sciences Ltd., London, UK). The search criteria were 30 ppm for precursor and 0.5 Da for fragments; ion scores no less than 20 at 95% confidence. Positive identification was matched to at least two unique spectra and had an E-value < 0.05. The false discovery rate (FDR) was set as less than 1%. Protein ratios were quantified based on the summed intensities of the spectrum matched. These ratios were log_2_ transformed. Student’s *t*-test and Benjamini and Hochberg correction were performed on the transformed ratio [18]. iTRAQ ratios that fulfilled the 1.3 fold criteria were considered significant. Proteins with expression ratios below 0.77 were considered to be down-regulated while those above 1.3 were considered up-regulated.

### 2-DE Western blot analysis

2-DE Western blot analysis for protein samples of MM and SF was performed to confirm the expression and distribution of the actin isoforms on 2-DE gels following the described protocol by Chandramouli et al. [9]. One hundred µg of protein were subjected to IEF on 7 cm IPG strips (Bio-Rad, Hercules, CA, USA), and separated on 12.5% SDS-PAGE. The proteins were transferred to PVDF membrane (Millipore Corporation, Bedford, MA, USA) and incubated (1:2000) with anti-actin clone C4 mouse monoclonal antibody (Millipore, Billerica, MA USA) for 14-16 hr at 4^o^C. The membranes were then incubated with corresponding HRP secondary antibody followed by detection using a WesternBright ECL detection kit (Advansta, Menlo Park, CA, USA).

## Results

### Comparison of protein and phosphoprotein spots in 2-DE gels of male and female worms

**Figure 2:**
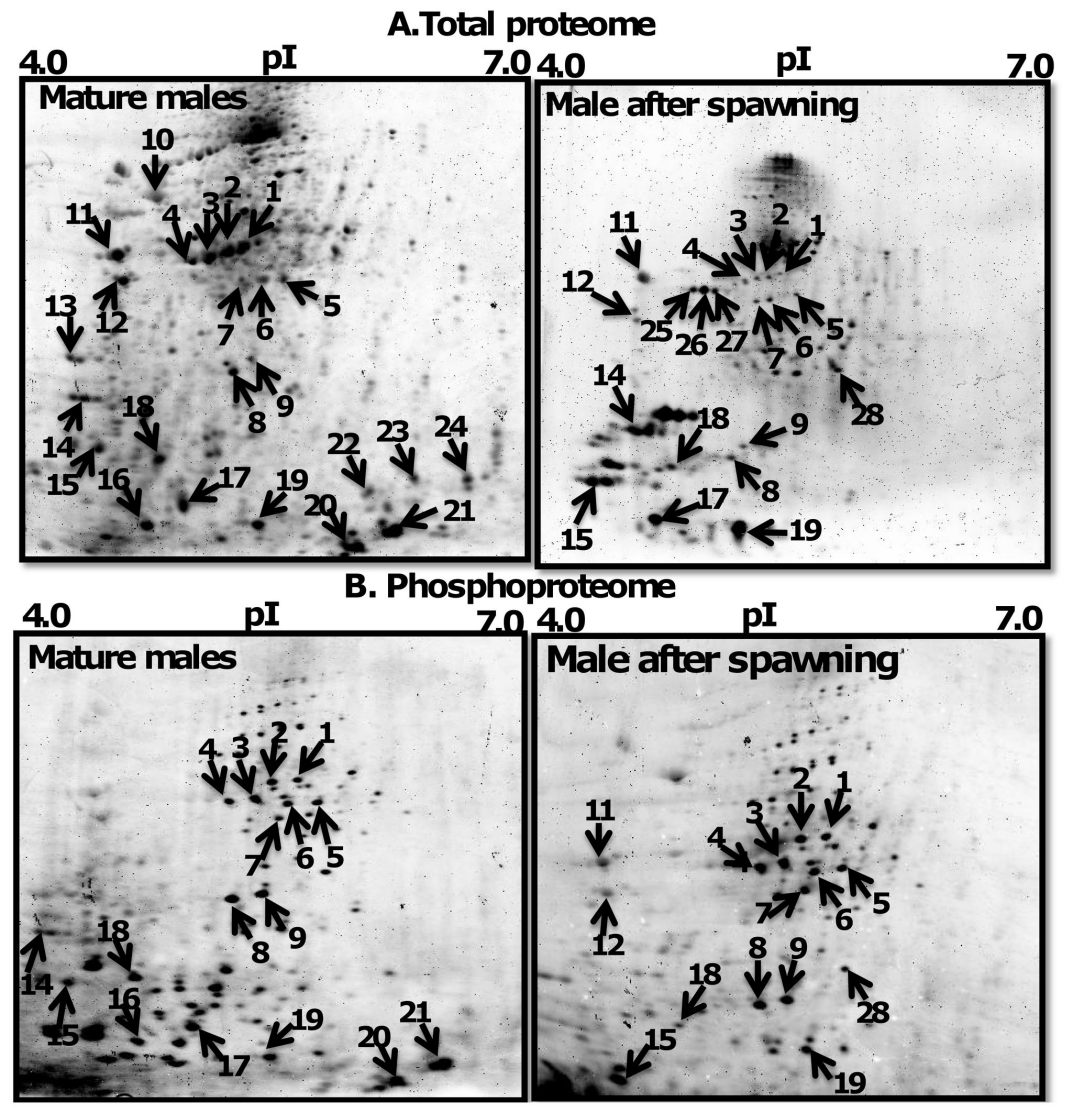
Proteome of mature males and males after spawning. 2DE gels stained with Sypro Ruby dye to detect total protein (upper panel) and Pro-Q Diamond dye to detect phosphoproteins (lower panel). Protein spots marked with arrow were identified by mass spectrometry.

**Figure 3:**
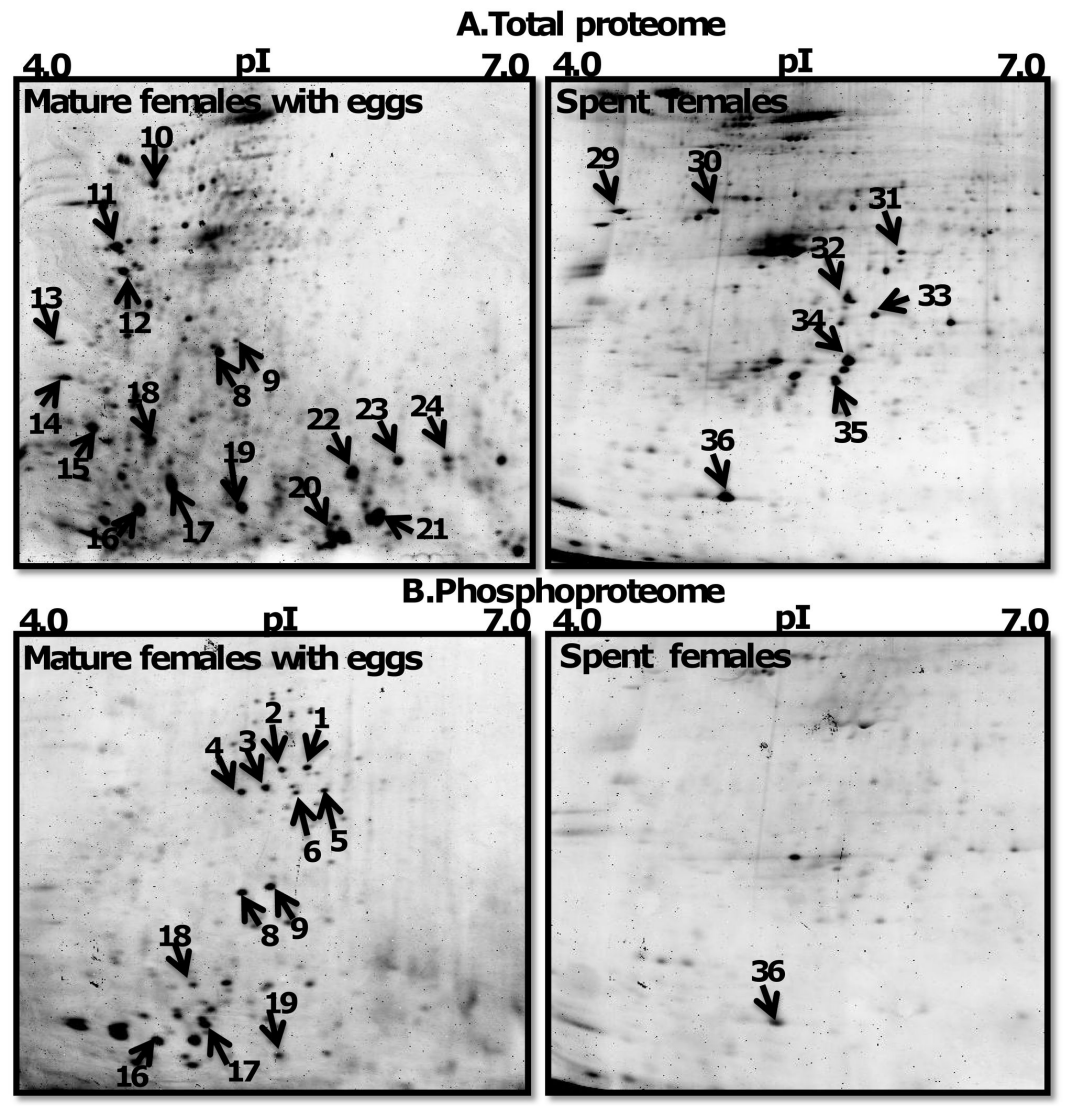
Proteome of mature females and spent females. 2DE gels stained with Sypro Ruby dye to detect total protein (upper panel) and Pro-Q Diamond dye to detect phosphoproteins (lower panel). Protein spots marked with arrow were identified by mass spectrometry.

**Figure 4:**
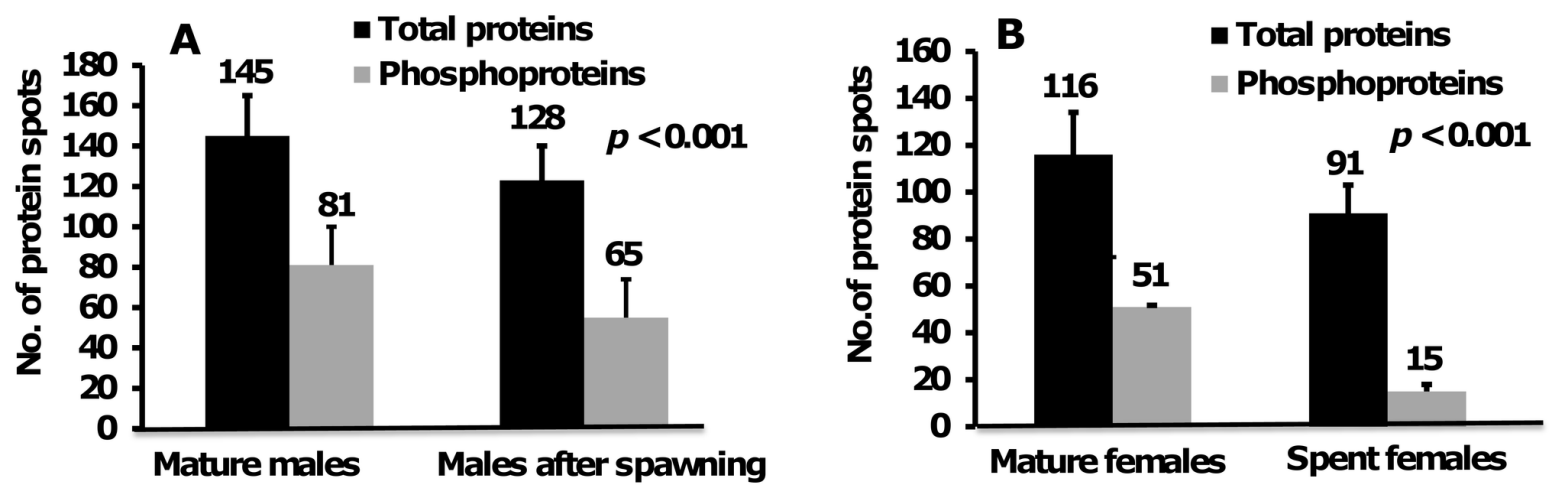
The number of protein and phosphoprotein spots detected in (a) mature males (MM), males after first spawning (MAS) and (b) mature females (MF), and spent females (S) of *N* *. arenaceodentata* . The protein spots were determined by a paired t-test with p-value (p < 0.001) using PDQuest software (version 8.0). Each error bar represents the mean (SD) number of proteins from three replicate gels.

Proteome and phosphoproteome 2-DE maps of MM, MAS, MF, and SF are shown in Figure 2 and Figure 3, respectively. There were 145, 128, 116, and 91 abundant protein and 81, 65, 51, and 15 phosphoprotein spots detected in MM, MAS, MF, and SF, respectively (Figure 4A and B). MM had more protein spots than other reproductive stages. For example, MAS, MF, and SF proteomes had 17 (11.7%), 29 (20%) and 54 (62.7%) fewer spots than MM proteome, respectively. MAS, MF, and SF phosphoproteomes had 16 (19.7%), 30 (37%) and 66 (81.5%) fewer spots than MM proteome respectively. The number of protein and phosphoprotein spots was less in SF than in MF, with 21% (25 spots) and 70% (36 spots), respectively.

### Identification of abundant protein spots

The reproducible abundant spots selected for protein identification are marked on 2-DE gels as shown in Figure 2 and Figure 3. In total, 36 proteins, of which 19 were phosphoproteins, were identified and listed in Table 1. The majority of the proteins appeared to be isoforms of the same protein. Several protein spots were identified either as myosin (spots 1-7, 12, 14) or non-muscle actin II (nm-ACT) (spots 15, 18, 19, 28, 25-27, 31-33 35) and actins (ACT) (spots 16, 22, 34, 17, 20-24). Spots 8 and 9 were identified as intermediate filament (IF) and spot 30 as beta-tubulin (β-TUB). Protein spots 10 and 29 were identified as catalytic enzymes such as glycoside hydrolase (GH) and protein disulfide isomerase (PDI), respectively. Spots 11 and 13 were identified as signaling regulatory molecules such as 14-3-3 and calmodulin (CaM). Spot 36 was identified as ubiquitous intracellular ferritin protein (FP).

**Table 1:** Proteins identified during reproductive stages of male and female worms of *Neanthes arenaceoentata *.

**Spot No.**	**Acc No. ^a^**	**NCBI accn.no**	**Protein name ^b^**	**MW(kDa)^c^ Obs./Theo.**	**pI^c^ Obs./Theo.**	**PM/SC (%)**
1-7*	isotig16356_3	gi|5817598	myosin heavy chain	32/50	4.9/5.4	2/15
8*, 9*	isotig00484_1	gi|633240	intermediate filament protein	67/30	5.4/4.9	4/11
10	GH8N3EB01BBNZO_5	gi|125975073	glycoside hydrolase	74/60	5.1/5.0	2/10
11	contig05426_2	gi|219806586	14-3-3	170/150	4.5/4.5	2/20
12	contig05426_2	gi|219806586	tropomyosin	15/35	4.5/4.5	2/20
13	isotig05673_4	gi|74025586	calmodulin	17/25	4.3/4.0	2/11
14*	isotig01747_2	gi|313677924	myosin light chain	17/23	4.3/4.2	2/14
15*,18*,19*,25-27, 28*, 31-33 35	isotig01855_7	gi|312861909	non-muscle actin II	42/25	5.3/4.5	2/16
16*, 22, 34	contig19579_5	gi|167683056	actin	21/20	5.5/5.0	3/41
17*,20*,21*-24	isotig14722_2	gi|50593062	beta-actin	16/22	5.4/5.4	3/20
29	isotig09632_9	gi|126697420	protein disulfide isomerase	57/60	4.5/4.5	2/13
30	isotig14472_10	gi|162138821	beta-tubulin	50/52	4.8/5.2	8/22
36*	contig18171_2	gi|212675249	ferritin-like protein	17/22	4.8/5.2	2/8

a) Accession numbers are in-house built transcriptome sequences of *Hydroides* b) For positive identification, the score had to be over the significance threshold level (p<0.05). c) Observed (Obs.) MW and pI values were estimated from 2-DE gels and theoretical (Theo.) PM: number of peptides matching the protein sequence; SC: sequence coverage. * phosphoproteins.

### Commonly expressed and specific sets of proteins in MM, MAS, MF, and SF worms

Figures 2 and 3 (upper panels spots marked with arrow) show common or specific expressed abundant total proteins spots identified as MS, MAS, IFs, GH, 14-3-3, tropomyosin (TM), CaM, myosin light chain (MLC) and ACT. These were expressed in MM, MAS, and MF but not SF. ACT isoforms, such ACT and β-ACT, exhibited specific expression in MM, whereas protein spots 25-28 showed specific expression to MAS. nm-ACT was present in both MM and MAS. Myosin heavy chain (MHC) (spots 1-7), 14-3-3 (spot 11), and TM (spot 12) were down-regulated in MAS (Figure 2). MF and SF proteome showed a lower degree of similarity in protein expression pattern. All other proteins were specifically expressed either in MF or SF with the exception of nm-ACTs which were present in both stages. IFs, GH, 14-3-3, TM, CaM, MLC, β-ACT, and ACT were specifically expressed in MF, whereas PDI, β-TUB, and FP were specific to SF (Figure 2). In contrast, males before and after spawning showed a higher degree of similarity in patterns. Both MM and MAS worms shared 21 (58%) identified proteins such as MLC, IFs, GH, 14-3-3, TM, CaM, MLC, and ACT isoforms (Figures 2 and 3 Left panels). Since MHC proteins (spots 1-7) were not detected in MF, but their phosphorylation level was noticeable in their respective ProQ Diamond 2-DE gels, it is possible that MSH proteins were down-regulated in MF (Figure 3 Left panel).

### Protein phosphorylation dynamics among MM, MAS, MF proteome

**Figure 5:**
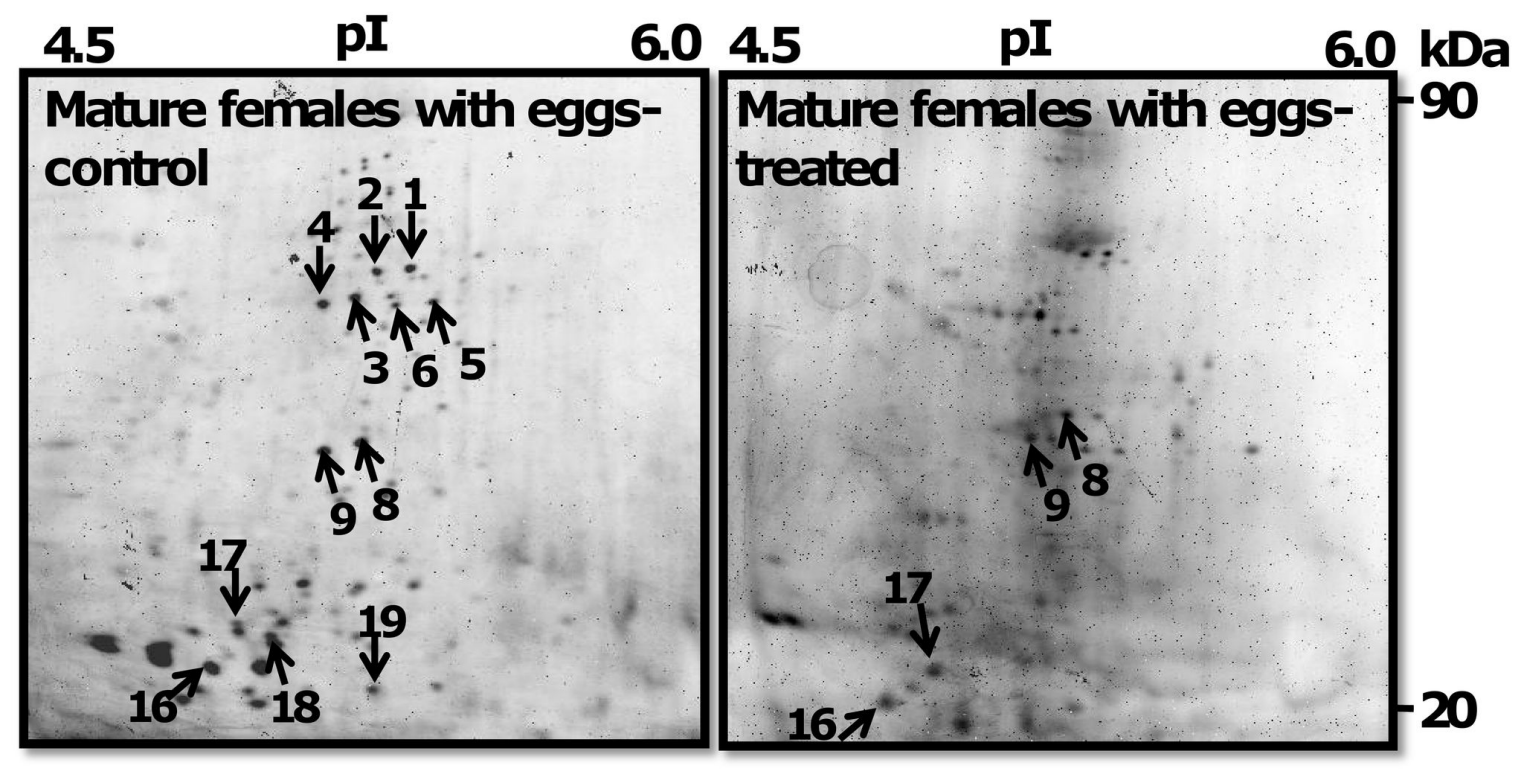
Mature female’s 2-DE gel treated with phosphatase showing dephosphorylated spots. 300 µg proteins were incubated without (control) or with (treated) 400 U ƛ-PPase and separated by 2-DE (pH range 4–7). Phosphorylated proteins (arrow heads) were detected by Pro-Q Diamond phosphoprotein stain. The treated gel shows the spot pattern in the presence of ƛ-phosphatase. Spot numbers are corresponding to proteins listed in Table 1.

MHC, IFs, and MLC were frequently phosphorylated in MM, MAS, and MF. MHC, ACT, and β-ACT showed specific phosphorylation to MM, whereas phosphoprotein nm-ACT (spot 28) was specific to MAS. The phosphoproteome pattern between MF and SF was different. Twelve proteins or isoforms of the same protein, such as MHC, IFs, GH, 14-3-3, TM, CaM, MLC and ACT isoforms, were phosphorylated in MF, whereas only FP was phosphorylated in SF. In contrast, MM and MF were similar in phosphorylation patterns. Both male stages shared 12 (63%) phosphoproteins such as MLC, IFs, and ACT isoforms. Protein spots were treated with phosphatase to validate the specificity of phosphoproteins detected on 2-DE gels. The enzymatic treatment resulted in either down-regulation or loss of phosphorylation of protein spots (Figure 5). MHC (spots 1-6) and nm-ACTs (spots 18, 19) were dephosphorylated and IFs (spots 8, 9), ACT (spot 16), and β-ACT (spot 17) phosphorylation was down-regulated upon phosphatase treatment of ProQ Diamond stained gels (Figure 5 right panel). These results validate the specificity of the phosphoprotein specificity of ProQ Diamond dye.

### Differential proteins identified in MM and MF by gel-free quantitative proteomics

Threshold (E<0.05 and 1% FDR) and normalization parameters were applied for iTRAQ data analysis in order to eliminate the false positive protein identifications. Proteins were quantified using Student’s *t*-test followed by Benjamini and Hochberg [18] correction and fold change cutoff for all iTRAQ ratios. In total, 19 differentially expressed proteins were identified, of which 10 were up-regulated and 9 were down-regulated in MF when compared with those in MM. Table 2 lists all the proteins which showed statistically significant expression changes along with their fold-change values. Table S1 provides the raw MS data of all the proteins identified. Six myosin proteins including TP, MHC, troponin C (TC), and paramyosin (PM) were down-regulated in MF worms. Two translation related proteins, such as ribosomal protein S2 (RP2) and elongation factor 1-alpha (EF1), were up-regulated. Six uncharacterized or partially characterized proteins were up-regulated. Four proteins, such as guanine nucleotide-binding protein (G-protein) and TUB, were also up-regulated, whereas ATP synthase and ACT were down-regulated (Table 2).

**Table 2:** Differential expressed proteins in mature male and female worms of *Neanthesarenaceodentata* .

***H. elegans* Accession no. ^a^**	**Protein name**	**Match to NCBI**	Proteins matched	Mean protein score	Mean sequence coverage (%)^b^	**female/male (116/114, 117/115)**	
						**FC** ^c^	**t-test**
***cellsignaling***							
isotig13580_74-793	Guanine nucleotide-binding protein G (i) subunit alpha	P30682.3	1	54	4.6	1.4	2.19E-02
isotig00463_289-948	ATP synthase, mitochondrial F1 complex	gb|EDL84889.1	2	108	13.9	0.7	0.23083
**structure and integrity**								
GF03LTE04JAQDB_8	tubulin, alpha 2 isoform 1	gi|320167203	4	137	21.2	1.5	2.41E-05
isotig17354_634-2	actin	ABU86741.1	44	565	45.6	0.6	5.84E-03
**translation**								
GF03LTE04ICYEJ_4	ribosomal protein S2	XP_781777.1	1	54	9.1	1.6	3.95E-03
GF03LTE04IF8R7_6	elongation factor 1-alpha	BAH28891.1	4	180	20.8	1.4	1.35E-02
**muscle contraction**								
contig03461_210-599	tropomyosin	gb|AAR87375.1	4	127	23.5	0.5	1.5E-05
GH8N3EB02FHJER_9	troponin C	BAB18897.1	4	88	10.2	0.7	3.89E-01
GGB57GC02DLEVO_4	paramyosin	BAJ61596.1	4	59	9.9	0.6	2.82E-03
GG7OGXE02D5NOV_5	myosin heavy chain	gb|ADU19853.1	4	74	6.4	0.7	5.94E-03
isotig02557_341-1315	myosin II heavy chain	gb|AAD13782.1	8	104	8.6	0.7	3.07E-02
isotig04410_268-681	myosin heavy chain, muscle isoform	XP_393334.4	6	47	6.5	0.5	5.14E-02
**unknown function**								
isotig13361_1568-210	GL12416-(protein_coding_gene)	XP_002019473.1	9	307	16.2	1.3	1.38E-03
isotig05207_82-1074	predicted protein	XP_001630154.1	5	202	14.8	1.4	1.82E-03
GGB57GC02EB6GL_9	GL12416	XP_002019473.1	4	121	26.4	1.4	2.02E-03
contig01907_1377-25	GL12416-like	XP_002731055.1	8	295	16.8	1.4	2.39E-03
isotig02084_1424-69	predicted protein	XP_001636867.1	7	296	17.2	1.3	2.81E-03
GH8N3EB02JDZSK_10	hypothetical protein	XP_002599176.1	1	54	15.1	1.4	2.31E-02
GG7OGXE02EAQVW_3	unnamed protein product	CAF95346.1	9	158	46.3	0.7	1.57E-02

FC: Fold change (*p*<0.05). ^a^ Accession nos. refers to contig no. in *Hydroides* transcriptome database. ^b^ Estimate only, based on a match to an EST which may represent an incomplete sequence. ^c^ Protein ratios in three biological replicates with <0.77 down-regulated ; > 1.3 up-regulated.

### Validation of abundant actin isoforms on 2-DE gels of MM and SM

**Figure 6:**
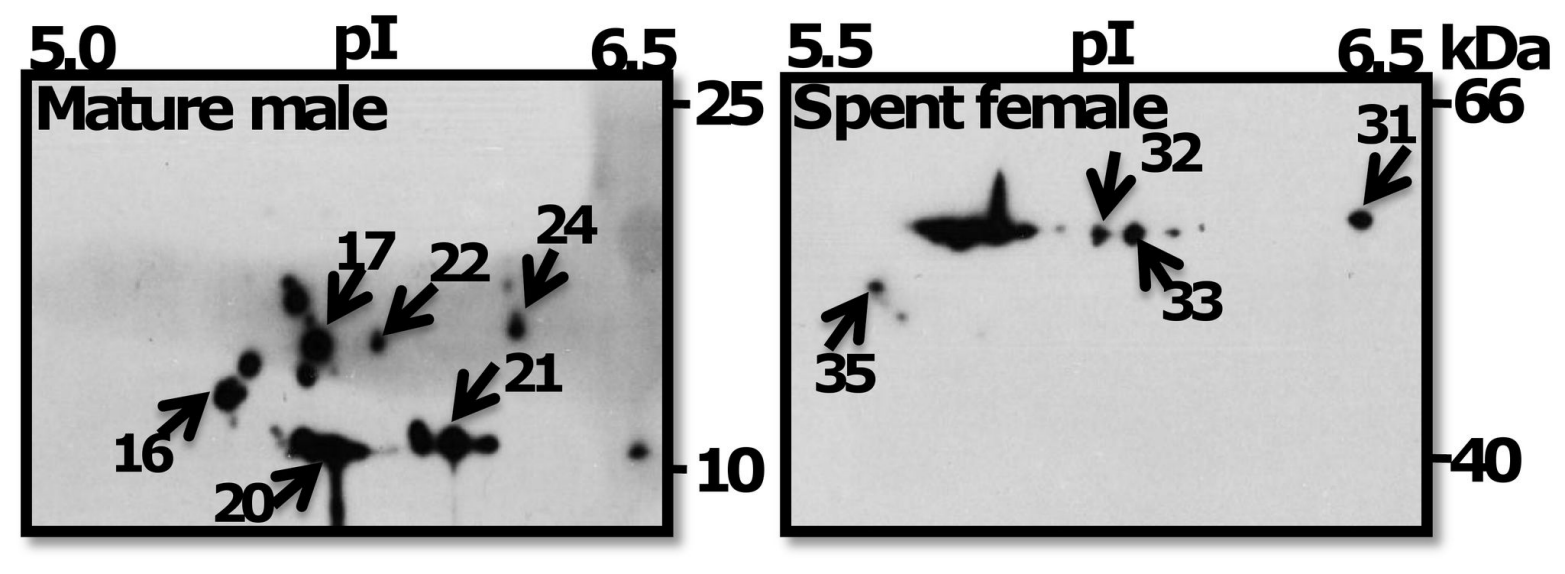
A close view of two-dimensional Western blot showing distribution of actin isoforms in mature male and spent female of *N* *. arenaceodentata* . Spot numbers are corresponding to protein spots marked in Figures 2 and 3. Corresponding proteins are listed in Table 1.

Commercial antibodies against polychaetes proteins are not available. Fifty two percent of the identified proteins were isoforms of ACT and showed different molecular mass (Mr) and isoelectric point (pI) values. Conserved protein actin was used to validate its abundant occurrence of isoforms on-2-DE gels. ACT (spots 16, 21, 22) and β-ACT isoforms (spots 17, 20, 24) had MW 25-10 and pI values 5.0-6.0 (Figure 6; left panel) in MM. SF showed specific isoforms of nm-ACTs (spots 31-33, 35) with Mr and pI valves ranged from 66–40 and pI values 5.5-6.5, respectively (Figure 6; right panel). Although ACT is highly conserved and expressed in male and female worms, the occurrence of different sets of its isoforms in MM and SF indicates the different functional and structural roles during reproduction in *Neanthes*.

## Discussion

### Proteomic changes in males before and after spawning

No previous studies have been done to distinguish molecular events during unusual reproductive behavior in male and female worms of polychaetes. This proteomic study provides molecular insights in protein expression pattern and their phosphorylation status during the reproductive period. Growth is usually measured by the number of segments in *Neanthes* [2]. The new segments may be added even 10 days before spawning. The occurrence of higher number of proteins in mature males before spawning may be attributed to a high demand of protein synthesis that is probably required for body growth and segment formation. In MM, the specific expression of ACT and nm-ACTs isoforms before and after spawning indicates their different functional and structural roles during reproductive development. For example, after fertilization the male undulates his body continuously to pump clean water and to expel metabolic wastes while incubating developing embryos [3], the nm-ACTs isoforms possibly facilitate the morphological movement of his body. The down-regulation of myosin and 14-3-3 proteins after spawning may indicate the sign of ageing which results in weakening of the body. MM fights off both sexes by utilizing most of his energy to safeguard the eggs during fertilization and development [2]. In MM, the specific expression of GH might facilitate the energy production from glycolytic pathway and its subsequent feedback control by CaM signaling molecules. GH is the major catalytic enzyme for the synthesis and breakage of glycosidic bonds [19]. The CaM is a multifunctional messenger protein that transduces calcium signals and interacts with various target proteins to mediate many biological processes [20].

### Protein Phosphorylation Dynamics in Males before and after Spawning

The proteomic changes that render mature males able to fertilize the eggs have been correlated with the changes in phosphorylation of several proteins. In a previous study, we identified tyrosine/serine phosphorylation of proteins in early larvae of *Neanthes* [9]. Identification of 19 phosphoproteins that exhibited gender/ stage specific phosphorylation suggests that the proteins, which influence reproductive changes, may be regulated by its phosphorylation changes (e.g. phosphorylation of specific set of proteins). For instance, phosphorylation of MLC and ACT isoforms were specific to MM, whereas 14-3-3 and TM showed specific phosphorylation after spawning. Despite these differences in specific phosphorylation, both MM and MAS worms shared 12 (63%) phosphoproteins (MLC, IFs, and ACT isoforms) that did not change expression. These common phosphoproteins, expressed before and after spawning, were probably required for the housekeeping functions during reproduction of the male [12]. The phosphorylation of MLC displays multi-functions in muscle tissues as well as non-muscle cells [21,22]. For example, cAMP-dependent kinase phosphorylates regulatory light chain isoform of MLC and modulates the kinase activity [23]. Transcripts of MLC and TM were shown to be involved in muscle development in competent larvae of *H. elegans* [24]. In previous study, we showed that the abundant expression of Ca^2+^/CaM dependent MLC kinase is crucial to larval settlement of the barnacle *Balanusamphitrite* [25]. In polychaete *H. elegans*, CaM gene was abundantly expressed in growth zones, branchial rudiments, and collar regions of competent larvae and juveniles [26]. The phosphorylation of IM and ACT isoforms may play a critical role in regulating the function of actin filaments of muscle and non-muscle cells [27]. Based on these findings, we deduced that phosphorylation of myosin and cytoskeleton proteins are an essential process that may influence developmental and reproductive changes in *Neanthes*.

### Changes in protein expression and phosphorylation in spent female

The body muscles of the female are reabsorbed to supply the nutrients for the large yolky eggs, which results in a loss of ~75% of her body weight [2]. The decrease in protein (21%) and phosphoprotein spots (70%) followed by low levels of phosphorylation may be attributed to protein degradation that had lead to the loss of body weight after egg laying [3]. The stress response (PDI) and hence metabolism (FP) proteins may be required to evade the immune system in response to weakening of body. The complete down-regulation of ACT isomers and IFs in SF may be attributed to the degenerating architecture of the cytoskeleton, which subsequently leads to breakdown of cellular processes. Myosins composed of actin-dependent molecular motor proteins are responsible for actin-based motility in skeletal muscle [28]. All eukaryotic cells composed of myosin isoforms have specialized functions in specific cell types such as muscle [29]. MHC, MLC, TM, TC, and PM proteins are highly conserved motor proteins and contribute to muscle contraction and motility processes [30]. Down-regulation of myosin in spent females may have affected the muscular contraction and thereby reduced their locomotive ability [2]. Furthermore, myosin function in cellular pathways is mediated by its interaction with specific proteins and is controlled by regulatory signals and protein phosphorylation [23,28]. For example, the contraction of skeletal and cardiac muscles is regulated by the coordinated interaction of myosin filaments with regulatory molecules such as Ca^2+,^ CaM, TC, and ACT that often act as an ON/OFF switch that activates or deactivates muscular contraction [31–33]. The most important finding of this study is the dephosphorylation reductions of ACT and MHC isoforms in SF. MF exhibited a high degree of phosphorylation of ACT and MHC isoforms whereas only FP was phosphorylated in SF. Furthermore, MF may have synthesized most of the abundant proteins that are subsequently phosphorylated. These proteins may serve as an energy source for maturing eggs. These results indicate that phosphorylation or dephosphorylation of specific proteins influences reproductive changes in females.

### Gender specific differential expression of proteome in MM and MF worms

The morphological and behavioral difference such as egg bearing female and egg incubating males may be influenced by differences in their proteome expression patterns that play an important role in reproduction [12]. Proteomes of MM and MF were less divergent before and after fertilization with the exception of changes in MHC (spots 1-7) and phosphorylation of ACT isoforms (spots 15, 20, 21) in 2-DE profiles. Similarly, MM and MF revealed similar trend of expression pattern of phosphoproteome. We believe that prior to spawning immature MM and MF exhibit common morphological features that are associated with the same set of proteome. In previous study [9], we proposed that 2-DE proteome analysis of whole worms increases sample complexity and thus prevents identification of low abundant proteins [34,35]. In addition, 2-DE proteomics generates proteome profiles that only represent highly abundant proteins [36,37]. These limitations accounted for less divergent 2-DE proteome profiles of MM and MF worms. To obtain comprehensive proteomics changes between MM and MF worms, we used quantitative proteomics that led to identification of 19 differentially expressed proteins. MM and MF worms showed differences in the levels of expression of proteins that are known to be involved in the cell signaling, structure and integrity, translation, and muscle contraction. These proteins possibly involved in signaling or sensing activities upon pairing and their subsequence reproductive changes between male and females. Down-regulation of MHC in MF is in agreement with the 2-DE proteomics data. In SF worms the lining of the digestive tract remains intact and body muscles undergo autolysis resulting in accumulation of phagocytized fragments of muscle tissue [38,39]. Eleocytes are produced in large numbers in both sexes shortly before and after the spawning [40]. Muscle fragments are used for the synthesis of vitellogenin, yolk precursor in oocytes [41]. In *N* *. arenaceodentata* , the lipid and protein needed for vitellogenin synthesis appear to be provided from muscle tissue degradation by coelomocytes [6]. Identification of several myosin fragments or isoforms in MM, MF, and MAS would further support this argument. The myosin function appears to be regulated in a number of ways; for example, by the presence or absence of phosphorylation, by Ca^2+^/CaM binding, and/or by binding to ACT, TC, and TM, which may synergistically modulate the reproductive events [31–33]. The up-regulation of G-proteins and translation proteins may indicate the synthesis and storage of new proteins that may be capable of converting chemical energy into mechanical work nutrients for the maturing eggs. Chaperone proteins (HSP90s) are flexible dimer ATPases that bind to receptors, transcription factors, and protein kinases are important for development and survival [42]. The reduced level of ATPase expression, which is capable of converting chemical energy into mechanical force, may also indicate the weakening of the female body. GL12416, hypothetical protein, and unnamed predicted proteins which were up-regulated in MF have no known function. 2-DE proteomics identified several proteins such as MHC, ACT, and TUB that constitutively expressed both in male and female worms. This pattern of observation is also consistent with the gel-free quantitative proteomics data suggesting that both techniques are complementary. The abundant expression of similar proteins in *P* *. vexillosa* and *H. elegans* may suggest that polychaetes maintain a pool of abundant proteins of housekeeping or regulatory functions [43,44].

### Validation of phosphoprotein spots and distribution of actin isoforms on 2-DE gel

Phosphatase treatment of protein spots 2-DE gel confirmed the phosphorylation of several proteins. This finding allowed us to speculate that MHC and ACT isoforms are probably involved in post-translational regulation, such as phosphorylation and dephosphorylation. The results of this experiment also confirmed the phosphorylation of several proteins on 2-DE gels. The results of 2-DE Western-blot confirmed the specific expression patterns of different isoforms in male (ACTs) and female (nm-ACTs) worms. These results suggest that although ACT is highly conserved in male and females, isoforms of certain ACT may play the same or different functional roles in modulating reproductive behavior in polychaetes. Overall, the validations experiments supports the hypothesis that protein modification (phosphorylation) dynamics influences reproductive pattern by activating (phosphorylation) or deactivating (dephosphorylation) protein’s function.

## Conclusion

We demonstrated gel-based and gel-free proteomic methods are feasible to document the proteomic differences between male and female worms before and after spawning of the polychaete worm *Neanthes*. The varieties of isoforms of myosin’s and actins may interact each other and influence the behavioral and morphological differences between sexes. The identified proteins involved in muscular development, cell signaling, structure and integrity, and translation were differentially expressed and probably regulate reproductive behavior. The proteome dataset of the male and female worms shall be an important resource for further understanding the unusual reproductive pattern in *Neanthes* *acuminata* complex in which the female reproduces once and the male is capable of reproducing many times [3].

## Supporting Information

Table S1 **Raw MS data of all the proteins identified by gel-free quantitative proteomics.**
(XLSX)

## References

[1] Glasby CJ (2005) Polychaete distribution patterns revisited: an historical explanation. Mar Ecol 26: 235-245. doi:10.1111/j.1439-0485.2005.00059.x.

[2] Reish DJ (1957) The life history of the Polychaetous Annelid *Neanthes caudata* (delle Chiaje), including a summary of development in the family Nereidae. Pac Sci 11: 216-228.

[3] Reish DJ, De Callibus K, Dewar J, Bube C (2009) Reproductive longevity in two species of polychaetous annelids. Zoosymposia 2: 391–395.

[4] Reish DJ, Gerlinger TV (1997) A review of the toxicological studies with polychaetous annelids. Bull Mar Sci 60: 584–607.

[5] Neave MJ, Streten-Joyce C, Nouwens AS, Glasby CJ, McGuinness KA et al. (2012) The transcriptome and proteome are altered in marine polychaetes (Annelida) exposed to elevated metal levels. J Proteomics 75: 2721-2735. doi:10.1016/j.jprot.2012.03.031. PubMed: 22484056. 22484056 10.1016/j.jprot.2012.03.031

[6] Lee RF, Walker A, Reish DJ (2005) Characterization of lipovitellin in eggs of the polychaete *Neanthes arenaceodentata* . Comp Biochem Physiol B Biochem Mol Biol 40: 381–386. PubMed: 15694585. 10.1016/j.cbpc.2004.11.002 15694585

[7] Rosen G, Miller K (2011) A post exposure feeding assay using the marine polychaete *Neanthes arenaceodentata* suitable for laboratory and in situ exposures. Environ Toxicol Chem 30: 730–737. doi:10.1002/etc.438. PubMed: 21298715. 21298715 10.1002/etc.438

[8] Winchell CJ, Valencia JE, Jacobs DK (2010) Confocal analysis of nervous system architecture in direct-developing juveniles of *Neanthes arenaceodentata* (Annelida, Nereididae). Front Zool 7: 17. doi:10.1186/1742-9994-7-17. PubMed: 20553614. 20553614 10.1186/1742-9994-7-17 PMC2909921

[9] Chandramouli KH, Reish D, Qian PY (2012) Gel-based and gel-free identification of proteins and phosphopeptides during egg-to-larva transition in polychaete *Neanthes arenaceodentata* . PLOS ONE 7: e38814. doi:10.1371/journal.pone.0038814. PubMed: 22719953. 22719953 10.1371/journal.pone.0038814 PMC3376139

[10] Chandramouli KH, Sun J, Mok FSY, Liu L, Qiu JW et al. (2013) Transcriptome and quantitative proteome analysis reveals molecular processes associated with larval metamorphosis in the polychaete *Pseudopolydora vexillosa* . J Proteome Res 12: 1344-1358. doi:10.1021/pr3010088. PubMed: 23294167. 23294167 10.1021/pr3010088

[11] Blank M, Mikkat S, Verleih M, Bastrop R (2012) Proteomic comparison of two invasive polychaete species and their naturally occurring F1-hybrids. J Proteome Res 11: 897-905. doi:10.1021/pr200710z. PubMed: 22185356. 22185356 10.1021/pr200710z

[12] Cheng GF, Lin JJ, Feng XG, Fu ZQ, Jin YM et al. (2005) Proteomic analysis of differentially expressed proteins between the male and female worm of *Schistosoma japonicum* after pairing. Proteomics 5: 511-521. doi:10.1002/pmic.200400953. PubMed: 15700243. 15700243 10.1002/pmic.200400953

[13] Takemori N, Yamamoto MT (2009) Proteome mapping of the *Drosophila melanogaster* male reproductive system. Proteomics 9: 2484–2493. doi:10.1002/pmic.200800795. PubMed: 19343724. 19343724 10.1002/pmic.200800795

[14] Moreno Y, Geary TG (2008) Stage- and Gender-Specific Proteomic Analysis of *Brugia malayi* Excretory-Secretory Products. PLOS Negl Trop Dis 2: e326. doi:10.1371/journal.pntd.0000326. PubMed: 18958170. 18958170 10.1371/journal.pntd.0000326 PMC2569413

[15] Thiyagarajan V, Wong T, Qian PY (2009) 2D gel-based proteome and phosphoproteome analysis during larval metamorphosis in two major marine biofouling invertebrates. J Proteome Res 8: 2708–2719. doi:10.1021/pr800976u. PubMed: 19341272. 19341272 10.1021/pr800976u

[16] Zhang H, Wong YH, Wang H, Chen Z, Arellano SM et al. (2011) Quantitative proteomics identify molecular targets that are crucial in larval settlement and metamorphosis of *Bugula neritina* . J Proteome Res 7: 349-360. PubMed: 21090758. 10.1021/pr100817v 21090758

[17] Sun J, Zhang H, Wang H, Heras H, Dreon MS et al. (2012) First proteome of the egg perivitelline fluid of a freshwater gastropod with aerial oviposition. J Proteome Res 11: 4240-4248. doi:10.1021/pr3003613. PubMed: 22738194. 22738194 10.1021/pr3003613

[18] Benjamini Y, Hochberg Y (1995) Controlling the false discovery rate: a practical and powerful approach to multiple testing. J R Stat Soc B Stat Methodol 57: 289–300.

[19] Sinnott ML (1990) Catalytic mechanisms of enzymatic glycosyl transfer. Chem Rev 90: 1171-1202. doi:10.1021/cr00105a006.

[20] Stevens FC (1983) Calmodulin: an introduction. Can J Biochem Cell Biol 61: 906–910. doi:10.1139/o83-115. PubMed: 6313166. 6313166 10.1139/o83-115

[21] Wysolmerski RB, Lagunoff D (1990) Involvement of myosin light-chain kinase in endothelial cell retraction. Proc Natl Acad Sci U S A 87: 16–20. doi:10.1073/pnas.87.1.16. PubMed: 2296576. 2296576 10.1073/pnas.87.1.16 PMC53190

[22] Kolodney MS, Elson EL (1995) Contraction due to microtubule disruption is associated with increased phosphorylation of myosin regulatory light chain. Proc Natl Acad Sci U S A 92: 10252–10256. doi:10.1073/pnas.92.22.10252. PubMed: 7479762. 7479762 10.1073/pnas.92.22.10252 PMC40774

[23] Sohma H, Inoue K, Morita FA (1988) cAMP-dependent regulatory protein for RLC—a myosin kinase catalyzing the phosphorylation of scallop smooth muscle myosin light chain. J Biochem 103: 431–435. PubMed: 2839466. 2839466 10.1093/oxfordjournals.jbchem.a122287

[24] Wang H, Qian PY (2010) Involvement of a novel p38 mitogen activated protein kinase in larval metamorphosis of the polychaete *Hydroides elegans* (Haswell). J Exp Zool (Mol. Dev. Evol) 314B:390–402 10.1002/jez.b.21344 20535771

[25] Chen Z-F, Wang H, Matsumura K, Qian PY (2012) Expression of Calmodulin and Myosin Light Chain Kinase during Larval Settlement of the Barnacle *Balanus amphitrite* . PLOS ONE 7: e31337. doi:10.1371/journal.pone.0031337. PubMed: 22348072. 22348072 10.1371/journal.pone.0031337 PMC3278446

[26] Chen ZF, Wang H, Qian PY (2012) Characterization and expression of calmodulin gene during larval settlement and metamorphosis of the polychaete *Hydroides elegans* . Comp Biochem Physiol B Biochem Mol Biol 162: 113-119. doi:10.1016/j.cbpb.2012.04.001. PubMed: 22507549. 22507549 10.1016/j.cbpb.2012.04.001

[27] Gunning PW, Schevzov G, Kee AJ, Hardeman EC (2005) Tropomyosin isoforms: divining rods for actin cytoskeleton function. Trends Cell Biol 15: 333–341. doi:10.1016/j.tcb.2005.04.007. PubMed: 15953552. 15953552 10.1016/j.tcb.2005.04.007

[28] Buss F, Kendrick-Jones J (2008) How are the cellular functions of myosin VI regulated within the cell? Biochem Biophys Res Commun 369: 165-175. doi:10.1016/j.bbrc.2007.11.150. PubMed: 18068125. 18068125 10.1016/j.bbrc.2007.11.150 PMC2635068

[29] Sellers JR (2000) Myosins: a diverse superfamily. Biochim Biophys Acta 1496: 3-22. doi:10.1016/S0167-4889(00)00005-7. PubMed: 10722873. 10722873 10.1016/s0167-4889(00)00005-7

[30] Tyska MJ, Warshaw DM (2002) The myosin power stroke. Cell Motil Cytoskeleton 51: 1-15. doi:10.1002/cm.10014. PubMed: 11810692. 11810692 10.1002/cm.10014

[31] Schaub MC, Perry SV (1969) The relaxing protein system of striated muscle: resolution of the troponin complex into inhibitory and calcium ion sensitizing factors. Biochem J 115: 993–1004. PubMed: 4243353. 4243353 10.1042/bj1150993 PMC1185242

[32] Hartshorne DJ, Theiner M, Mueller M (1969) Studies on troponin. Biochim Biophys Acta 175: 320–330. doi:10.1016/0005-2795(69)90009-9. PubMed: 4238076. 4238076 10.1016/0005-2795(69)90009-9

[33] Greaser ML, Gergely J (1971) Reconstitution of troponin activity from three protein components. J Biol Chem 246: 4226–4233. PubMed: 4253596. 4253596

[34] Chandramouli KH, Mok FS, Wang H, Qian PY (2011) Phosphoproteome analysis during larval development and metamorphosis in the spionid polychaete *Pseudopolydora vexillosa* . BMC Dev Biol 11: 31. doi:10.1186/1471-213X-11-31. PubMed: 21612608. 21612608 10.1186/1471-213X-11-31 PMC3115903

[35] Chandramouli KH, Soo L, Qian PY (2011) Differential expression of proteins and phosphoproteins during larval metamorphosis of the polychaete *Capitella* sp. I. Proteome Sci 9: 51. doi:10.1186/1477-5956-9-51. PubMed: 21888661. 21888661 10.1186/1477-5956-9-51 PMC3180302

[36] Issaq H, Veenstra T (2008) Two-dimensional polyacrylamide gel electrophoresis (2D-PAGE): advances and perspectives. BioTechniques 44: 697-700. PubMed: 18474047. 18474047 10.2144/000112823

[37] Chevalier F (2010) Highlights on the capacities of gel-based proteomics. Proteome Sci 8: 23. doi:10.1186/1477-5956-8-23. PubMed: 20426826. 20426826 10.1186/1477-5956-8-23 PMC2873371

[38] Davis WR, Reish DJ (1975) The effect of reduced dissolved oxygen concentration on the growth and production of oocytes in the polychaetous annelid *Neanthes arenaceodentata* . Rev Int Oceanogr Med 37-38:3–16

[39] Marsden JR (1966) The coelomocytes of *Hermodice carunculata* (Polychaeta: Amphinomidae) in relation to digestion and excretion. Can J Zool 44: 377–389. doi:10.1139/z66-041.

[40] Fischer A, Rabien H (1986) Molecules and cellular functions driving oocyte growth in nereid annelids. Adv Invertebr Reprod 4: 195–205.

[41] Baert JL, Slomianny MC (1987) Heterosynthetic origin of the major yolk protein, vitellin, in a nereid, *Perinereis cultrifera* (Polychaete Annelid). Comp Biochem Physiol B 88: 1191–1199.

[42] Dilly GF, Young CR, Lane WS, Pangilinan J, Girguis PR (2012) Exploring the limit of metazoan thermal tolerance via comparative proteomics: thermally induced changes in protein abundance by two hydrothermal vent polychaetes. Proc Biol Sci 279: 3347-3356. doi:10.1098/rspb.2012.0098. PubMed: 22553092. 22553092 10.1098/rspb.2012.0098 PMC3385714

[43] Chandramouli KH, Zhang Y, Wong YH, Qian PY (2012) Comparative glycoproteome analysis: Dynamics of protein glycosylation during metamorphic transition from pelagic to benthic life stages in three invertebrates. J Proteome Res 11: 1330–1340. doi:10.1021/pr200982k. PubMed: 22111546. 22111546 10.1021/pr200982k

[44] Zhang Y, Sun J, Xiao K, Arellano SM, Thiyagarajan V et al. (2010) 2D gel-based multiplexed proteomic analysis during larval development and metamorphosis of the biofouling polychaete tubeworm *Hydroides elegans* . J Proteome Res 9: 4851-4860. doi:10.1021/pr100645z. PubMed: 20666481. 20666481 10.1021/pr100645z

